# Multiomics analysis elucidated molecular mechanism of aromatic amino acid biosynthesis in *Akebia trifoliata* fruit

**DOI:** 10.3389/fpls.2022.1039550

**Published:** 2022-11-08

**Authors:** Shengfu Zhong, Ju Guan, Chen Chen, Feiquan Tan, Peigao Luo

**Affiliations:** Key Laboratory of Plant Genetics and Breeding at Sichuan Agricutural University of Sichuan Province, College of Agronomy, Sichuan Agricultural University, Chengdu, China

**Keywords:** *Akebia trifoliata*, aromatic amino acids, multiomics, biosynthetic mechanism, whole genome duplication

## Abstract

*Akebia trifoliata* is a novel edible and healthy fruit. Here, we found that this fruit had the highest content of total free amino acids and three aromatic amino acids (AAAs) compared with the other popular fruits, and there was an obvious inverse relationship between AAA and flavonoid levels in various fruit tissues. Multiomics analysis revealed that the evolutionarily strengthened synthetic pathway of all three AAAs, the largely regulating ability conferred by *ASP5* in the arogenate pathway and the complementary phenylpyruvate pathway endorsed by *ADT* of both Phe and Tyr biosynthesis provided reasonable explanations for the high AAA content in the flesh of *A. trifoliata* fruit. Gene-specific expression could be the main reason for the inverse relationship between AAAs and flavonoids. This study will help us understand the metabolic mechanism of AAAs and to develop *A. trifoliata* as a fresh fruit crop and medicinal plant by molecular breeding strategies.

## Introduction

Amino acids are necessary structural units of all organisms and are very important nutrient materials for almost all life on earth. In addition, amino acids play a key role in various biological processes, such as ATP generation (Koopman et al., 2010), nucleotide synthesis (Laxman et al., 2013), redox balance (Buck et al., 2017), signaling pathways (Zhang et al., 2021) and cellular immunity (Kelly and Pearce, 2020). Individual amino acids can be created under non-biological chemical synthesis (Burton et al., 2012), but the ability to actively synthesize all or some amino acids is essential for all species, especially autotrophic organisms.

In plants, aromatic amino acids (AAAs), including tryptophan (Trp), phenylalanine (Phe) and tyrosine (Tyr), also serve as precursors of many secondary metabolites, such as flavonoids, alkaloids and hormones (Lynch and Dudareva, 2020). In humans, the accumulation of AAA metabolites produced by the gut microbial pathway can enhance immunity (Dodd et al., 2017). AAAs are also precursors of neurotransmitters such as dopamine, epinephrine, norepinephrine, serotonin and tryptamine (Gatellier et al., 2009). Unfortunately, most of animals have lost the ability to synthesize aromatic amino acids in the the process of evolution, especially higher animals (Costa et al., 2015). Therefore, AAAs and some of their derivatives, such as vitamins, are essential in the human diet for our survival and health, while plants are the good source of these nutrients.

All AAAs are derived from the shikimate pathway, which links carbohydrate metabolism to aromatic compound biosynthesis and is only found in microorganisms and plants (Tzin et al., 2009). At the same time, AAAs are also precursors of thousands of primary and specialized metabolites (Widhalm and Dudareva, 2015; Schenck and Maeda, 2018). For example, Trp is the precursor to auxin, alkaloids, indole glucosinolates and phytoalexins, while Tyr serves as the precursor for quinones, betalains, and isoquinoline alkaloids (Barros et al., 2016). However, the carbon flux forward to Phe is the major contributor to the high carbon flux in the shikimate pathway because it can produce more than 8000 secondary metabolites, such as anthocyanins, flavonoids, isoflavonoids, tannins, and volatiles (Maeda and Dudareva, 2012). Hence, further understanding the specific biosynthetic mechanism of AAAs in various species is valuable for both completely elucidating the AAA metabolic process in plants and finally finding new rich AAA food resources.

*Akebia trifoliata*, belonging to the Ranunculaceae family, is a woody perennial climbing vine (Li et al., 2010), and the fruit of *A. trifoliata*, also called augmelon, has increasingly attracted the attention of many consumers due to its abundant nutrients and health benefits (Yang et al., 2021). A recent study suggested that the largest commercial product of *A. trifoliata* should be as a new fresh fruit crop because its flesh contains a high content of free amino acids, especially AAAs and a very low content of flavonoids, while the peel has low free amino acid contents and high flavonoid contents (Guan et al., 2022). These properties also make *A. trifoliata* an ideal species for studying the molecular mechanisms of AAA metabolism. In addition, the artificial cultivation and improvement of *A. trifoliata* is still in its infancy. In order to better develop the augmelon fruit varieties, it is particularly important to analyze the main mechanism of the accumulation of aromatic amino acids in *A. trifoliata* fruit.

Multiomics, mainly consisting of genomics affording the basic information of all genes, transcriptomes containing the expression data of all functional genes and metabolomes giving the chemical traits of the final materials, is an effective and widely used method in the biological research field. In this study, we systemically characterized the profiling of genes, expressed sequences and final metabolites involved in AAA metabolism pathways of *A. trifoliata* fruit. In addition, we also compared the free amino acid content of *A. trifoliata* fruit with that of other popular fruits. Our main objectives were to understand the evolutionary mechanism at the genome level, the tissue-specific expression mechanism at the transcriptome level and the accumulation mechanism at the metabolome level and to develop strategies to further improve *A. trifoliata* as a new food resource.

## Materials and methods

### Plant materials

The clonal line “Shusen 1” of *A. trifoliata* was used for transcriptome sequencing and metabolome analysis in this study because the *A. trifoliata* reference genome was also produced by this line, and all the clonal lines were planted in Germplasm Nursery at Chongzhou Research Station (30°43’N, 103°65’E) of Sichuan Agricultural University. Three tissues (flesh, peel and seed) of *A. trifoliata* fruits at four developmental stages (young, enlargement, coloring and mature stages) (Yang et al., 2021) were randomly collected from 2-year-old plants, and three biological replicates were executed so that a total of 36 samples were used for RNA-seq and metabolite analysis. All fresh samples were immediately frozen in liquid nitrogen and then stored at -80°C.

### Determination of *A. trifoliata* fruit metabolites

Completely ripened fruits of *A. trifoliata* were utilized to determine the free amino acid contents, and four fruit varieties, Fuji apple (*Malus pumila*), Xianren banana (*Musa nana*), Fengshui pear (*Pyrus sorotina*) and Kyoho grape (*Vitis vinifera*), purchased from Ito Yokado Supermarket, were used as control samples of current popular fruit. A total of 15 flesh samples were separated from mature fruits of these 5 varieties, with three replicates for each variety. The content of 20 common free amino acids was determined by High Performance Liquid Chromatography (HPLC) method with minor modifications (Guan et al., 2022). In briefly, approximately 2 g of homogenized pulp was mixed with 1 mL of 15% salicylsulfonic acid. Then the homogenates were separated and determined using an Agilent 1260 Infinity HPLC system (Agilent Technologies Inc., USA) at a wavelength of 550 nm for 100 min. Seventeen amino acids (MembraPure, Berlin, Germany) were used as standard samples.

The total flavonoid contents of three tissues (flesh, peel, seed) from ripe augmelon fruit with three replicates were determinded by spectrophotometry according to the previously method (Guan et al., 2022). A mixture of 2 g of pulp with 10 mL of 70% aqueous ethanol solution was sonicated for 30 min (20 kHz, KQ-300DE, ShuMei). Then 10 mL of the 5 times diluted liquid was transferred into a 25-mL volumetric flask, and 2 mL of 5% NaNO_2_, 2 mL of 10% AlCl_3_, 6 mL of 1 M NaOH and 5 mL of 70% aqueous ethanol solution were sequentially added. This mixture was used to detect the absorbance at 510 nm using a spectrophotometer (UV-2401, Shimadzu Co., Japan); rutin was used as the standard to make the calibration curve.

Untargeted metabolite extraction and measurement were performed by the Metware Biotechnology Co. Ltd. (Wuhan, China). Each of above 36 frozen samples was analyzed using an LC-ESI-MS/MS system (HPLC, Shim-pack UFLC SHIMADZU CBM30A system; MS, Applied Biosystems 4500 Q TRAP). The analytical methods and conditions were as previously described (Chen et al., 2013). Briefly, each frozen sample was crushed using a mixer mill (MM 400, Retsch) with a zirconia bead for 1.5 min at 30 Hz. 100 mg powder was weighted in a 2.0 mL centrifuge tube and extracted overnight at 4 °C with 1.0 mL 70% aqueous methanol. The homogenate was centrifuged at 10,000g for 10 min and then the extracts were absorbed (CNWBOND Carbon-GCB SPE Cartridge, 250mg, 3ml; ANPEL, Shanghai,China) and filtrated (SCAA-104, 0.22 μm pore size; ANPEL,Shanghai, China) before LC-ESI-MS/MS analysis. The untargeted metabolome scans were controlled by Analyst 1.6 software The peak area of all mass spectra was integrated, and the mass spectra of the same metabolite in different samples were corrected integration base on the metware database and the Kyoto Encyclopedia of Genes and Genomes (KEGG) pathway database (Fraga et al., 2010).

### Gene characterization and coexpression analysis of AAA and flavonoid metabolism

The *A. trifoliata* genome assembly (Shusen 1) was downloaded from the Genome Warehouse in the National Genomics Data Center under accession number GWHBISH00000000, which is publicly accessible at https://ngdc.cncb.ac.cn/gwh. Each total ribonucleic acid (RNA) of above 36 frozen samples was isolated using TRIzol reagent (Invitrogen, Carlsbad, CA, USA) according to the manufacturer’s protocol, respectively. The transcriptome library with 240 bp inserts was prepared using an Illumina Kit and sequenced on an Illumina HiSeq™ 2500 (Illumina, Inc. 9885 Towne Centre Drive, San Diego, CA, USA), using paired-end technology. FASTX-Toolkit (v 0.0.13) (Gordon, Cold Spring Harbor, NY, USA) and Fastqc (Andrews, 2014) was used to obtain and check the clean reads by removing both adaptor and low-quality bases such as length less than 20 bp or N bases more than 10% and also by excluding short reads of less than 25 bp in length. The clean reads of the sequences were aligned to the *A. trifoliata* genome assembly by using HISAT v2.0.438, and the abundance of genes in different samples was computed with StringTie v1.2.3 (Haas et al., 2008). Gene expression levels were estimated in fragments per kilobase of transcript per million fragments mapped (FPKM) values using DESeq2 (Love et al., 2014). Gene expression in 4 stages and 3 tissue samples in *A. trifoliata* was evaluated with the WGCNA (Weight Gene Coexpression Network Analysis) R package v1.51 (Langfelder and Horvath, 2008) to identify modules of coexpressed genes with a soft threshold value (power) = 0.8 and cutHeight = 0.4. A total of 6434 expressed genes were obtained from all 36 samples by filtering out with FPKM < 1 or FPKM variance in the top 50%. Four development stages, three tissues, three AAAs and 15 major metabolic node flavonoids were used as the main traits analysis in the WGCNA. The correlation between the gene expression cluster modules and these objective traits was calculated by default parameters. Hub genes were screened by strongly associated with phenotypes with edge weights ≥ 0.30 and plotted by Cytoscape (Shannon et al., 2003). An integrated AAA and flavonoid biosynthesis pathway model with enzymes encoding genes and metabolites was manually constructed based on the above related KEGG pathways and previous reports (Yoo et al., 2013; Lynch and Dudareva, 2020). Cluster heatmaps of the FPKM values of the expressed genes and metabolite contents were plotted with TBtools (Chen et al., 2020). We predicted AAA and flavonoid biosynthesis- and metabolism-related genes according to KEGG functional gene annotations in the ‘phenylalanine, tyrosine and tryptophan biosynthesis’ (ko00400), ‘phenylalanine metabolism’ (ko00360) and ‘phenylpropanoid biosynthesis’ (ko00940) pathways and previous reports (Lin et al., 2015; El-Azaz et al., 2016; Qian et al., 2019; Barros and Dixon, 2020). WGD paralogs were identified through genome synteny analysis using TBtools v1.098769 with the default parameters (Chen et al., 2020).

### Cloning and subcellular location of key genes

Full-length *ASP5* (*EVM0012020*) cDNA was isolated from total RNA extracted from *A. trifoliata* seedlings by reverse transcription PCR using iScript Reverse Transcription Supermix (Bio-Rad) with specific primer pair 1 (**Table S1**). Then, cDNAs were subcloned into the PC2300-35s-eGFP vector (Biovector Co.,LTD) without a stop codon *via* homologous recombination cloning technology with specific primer pair 2 (**Table S1**). It was further fused to the N-terminus of the green fluorescent protein (eGFP) gene between the *BamI* and *SalI* sites under the control of the 35S promoter. The blank control *eGFP* and recombinant *eGFP*-*ASP5* plasmids were introduced into the *Agrobacterium tumefaciens* GV3101 (pSoup-19) strains, respectively. eGFP-tagged ASP5 proteins were expressed through agroinfiltration (OD600 = 0.5) in *Nicotiana benthamiana* leaves, as previously described (He et al., 2004). eGFP fluorescence was monitored 48 h after agroinfiltration using a laser scanning confocal microscope (Carl Zeiss LSM-800, Oberkochen, Germany). For eGFP detection, the excitation source was an argon ion laser at 488 nm, and emission was observed between 505 and 525 nm. Chloroplast autofluorescence was collected between 680 and 700 nm.

### Statistical analysis

The multiple comparison of free amino acid and total flavonoid contents between different fruits were determined with Duncan method using SPSS software version 22.0 (SPSS Inc., Chicago, IL, USA). Then the significant difference at P < 0.05 was indicated by lowercase letters. Because high-throughput metabolites of 36 samples were measured by untarget metabolome, the content of each metabolite was normalized into the percentage of relative content using the maximum content as 100%. Principal component analysis (PCA) was used to reflect the relative variation of the metabolite contents between different tissues and development stages. The differential metabolites between different groups of 36 samples were identified by orthogonal projections to latent structures-discriminant analysis (OPLS-DA) according to a fold change (FC) ≥ 2 or FC ≤ 0.5 and a variable importance in project (VIP) value ≥1 (Thévenot et al., 2015). Correlation coefficients and P values between the gene expression cluster modules and traits (tissue, stage, and metabolites) were directly calculated from WGCNA method by default parameters.

## Results

### Both the component and content of free amino acids in the flesh of *A. trifoliata* fruit

The flesh of mature *A. trifoliata* fruit has a delicious and sweet flavor and a soft juicy texture. We detected all 20 common free amino acids in the ripe flesh of *A. trifoliata* fruit and of four other common fruits: apple, banana, pear and grape (**Table 1**). In all five fruits, both asparagine (Asn) and glutamine (Gln) were the two richest amino acids, while aspartic acid (Asp) was the least abundant amino acid. Further comparison analysis found that the total content of 20 free amino acids (AAs) was highest (10103 ug/g) in augmelon and lowest (2707 ug/g) in apple. The concentration of the total AAs were significantly higher (P<0.05) in augmelon than the other fruits except for pear. In details, five amino acids (Phe, Trp, Tyr, cysteine (Cys), glutamic acid (Glu)) had significantly higher contents in *A. trifoliata* than in the other four fruits (**Table 1**). In particular, both the total contents (2095.99 ug/g) and the proportion (20.75%) of AAAs, including Phe, Tyr and Trp, were significantly higher in *A. trifoliata* fruit. The essential AAs of *A. trifoliata* fruit were also significantly higher than those of the other tested fruits. These results indicated that there could be a specific biosynthetic mechanism of AAAs in *A. trifoliata*.

**Table 1:** Contents of free amino acids in the flesh of *A. Trifoliate*.

Amino acids (AAs)	*A. trifoliata*	Apple	Banana	Pear	Grape
Alanine (Ala)	333.52 ± 64.07^a^	414.34 ± 130.08^a^	550.17 ± 166.44^a^	519.64 ± 181.93^a^	894.76 ± 368.45^a^
Arginine (Arg)	18.75 ± 6.95^b^	5.75 ± 1.20^ab^	61.38 ± 5.31^c^	5.45 ± 1.87^ab^	4.48 ± 0.93^a^
Asparagine (Asn)	3016.47 ± 354.83^b^	485.81 ± 132.22^a^	1229.54 ± 90.34^a^	2955.84 ± 192.31^b^	1142.75 ± 400.46^a^
Aspartic acid (Asp)	0.06 ± 0.01^ab^	0.17 ± 0.02^c^	0.02 ± 0.00^a^	0.08 ± 0.01^ab^	0.13 ± 0.05^bc^
Cysteine (Cys)	36.14 ± 15.49^b^	9.29 ± 2.86^a^	12.88 ± 3.10^a^	4.77 ± 1.90^a^	10.86 ± 2.83^a^
Glutamine (Gln)	2888.16 ± 351.32^b^	1154.79 ± 116.23^a^	2443.78 ± 172.52^ab^	2826.74 ± 844.98^b^	2418.6 ± 307.59^ab^
Glutamic acid (Glu)	836.17 ± 175.28^c^	298.71 ± 16.64^ab^	32.25 ± 1.98^a^	253.72 ± 44.15^ab^	348.94 ± 98.46^b^
Glycine (Gly)	48.17 ± 18.43^a^	10.99 ± 3.74^a^	326.19 ± 29.36^b^	13.44 ± 2.24^a^	41.17 ± 9.83^a^
Histidine (His)	137.75 ± 49.66^a^	4.55 ± 0.14^a^	1144.15 ± 178.43^c^	50.79 ± 11.81^a^	778.62 ± 87.4^b^
Proline (Pro)	595.5 ± 112.53^b^	70.4 ± 18.74^a^	275.18 ± 53.09^ab^	355 ± 182.16^ab^	326.3 ± 138.09^ab^
Serine (Ser)	31.17 ± 1.01^a^	30.25 ± 2.41^a^	26.21 ± 4.50^a^	1536.47 ± 491.78^b^	30.16 ± 1.92^a^
Threonine^d^ (Thr)	222.98 ± 10.21^a^	26.98 ± 4.73^a^	127.78 ± 8.89^a^	91.33 ± 30.20^a^	800.93 ± 133.56^b^
Valine^d^ (Val)	6.68 ± 3.34^a^	0.84 ± 0.29^a^	2.93 ± 0.78^a^	3.08 ± 0.16^a^	6.6 ± 1.70^a^
Methionine^d^ (Met)	58.67 ± 15.81^a^	10.23 ± 2.87^a^	372.5 ± 31.81^c^	199.32 ± 61.61^b^	55.02 ± 9.97^a^
Lysine^d^ (Lys)	20.49 ± 3.06^a^	3.29 ± 1.41^a^	82.35 ± 22.08^b^	3.02 ± 0.99^a^	5.68 ± 1.86^a^
Isoleucine^d^ (Ile)	5.35 ± 1.63^a^	0.73 ± 0.03^a^	30.09 ± 11.19^b^	17.64 ± 5.90^ab^	3.05 ± 0.58^a^
Leucine^d^ (Leu)	21.11 ± 7.18^a^	0.69 ± 0.33^a^	281.94 ± 98.47^b^	7.72 ± 2.76^a^	17.45 ± 3.42^a^
Tryptophan^d^ (Trp)	1756.44 ± 266.34^b^	175.79 ± 17.58^a^	578.71 ± 114.68^a^	398.53 ± 97.23^a^	588.41 ± 211.51^a^
Phenylalanine^d^ (Phe)	69.44 ± 1.81^c^	3.32 ± 1.04^a^	45.88 ± 3.45^b^	8.1 ± 3.23^a^	37.91 ± 5.16^b^
Tyrosine (Tyr)	270.11 ± 41.51^c^	4.57 ± 1.44^a^	157.74 ± 21.91^b^	15.26 ± 4.03^a^	14.3 ± 6.66^a^
Total AAs	10103.02 ± 522.33^c^	2706.92 ± 259.15^a^	7623.91 ± 255.43^b^	9250.66 ± 547.46^c^	7511.84 ± 359.89^b^
Aromatic AAs	2095.99 ± 245.55^c^ (20.75%)	183.68 ± 18.11^a^ (6.79%)	782.33 ± 136.03^ab^ (10.26%)	421.89 ± 98.88^b^ (4.56%)	640.62 ± 205.26^ab^ (8.53%)
Essential AAs	2161.17 ± 250.06^c^ (21.39%)	221.88 ± 20.86^a^ (8.20%)	1522.17 ± 250.87^b^ (19.97%)	728.74 ± 148.2^a^ (7.88%)	1515.05 ± 99.19^b^ (20.17%)

^a, b, c…^-Different lowercase letters in the same column show significant differences between different fruits at the p < 0.05 level.

The data are presented as the mean ± standard error (ug/g FW), and the percentage number (%) represents the proportion of amino acids among the total amino acids.

^d^Essential amino acids.

### Metabolome profile of three *A. trifoliata* fruit tissues at four different stages

**Figure 1:**
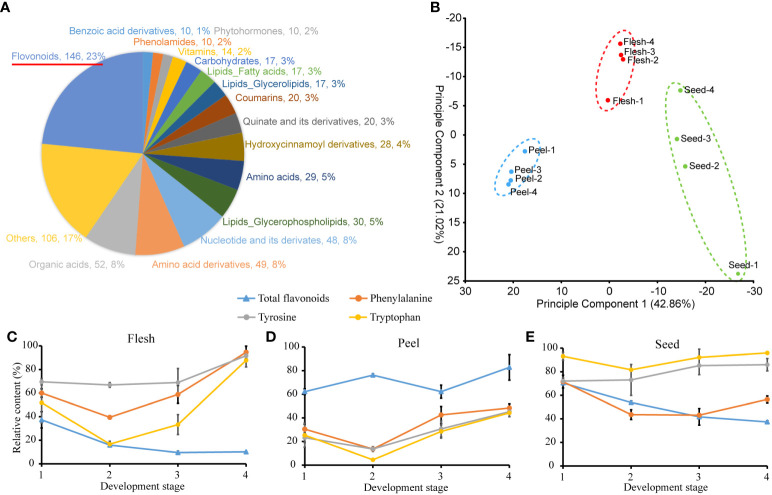
Metabolite measurements of flesh, peel and seed tissues at 4 developmental stages. **(A)** Classification of metabolites. **(B)** Plot of principal component analysis of metabolite contents between 3 tissues and 4 development stages. **(C–E)** Relative contents of aromatic amino acids and total flavonoids in different stages and tissues. The maximum content of the given metabolite among 36 samples is as 100%.

To further characterize the changes in metabolite abundance during fruit maturation, the metabolome of three *A. trifoliata* fruit tissues at four different stages was measured and analyzed. A total of 581 metabolites were detected from all tested samples (**Figure 1A** and **Table S2**), among which the number of flavonoids mainly derived from Phe was the highest (146). PCA analysis showed that variation of metabolite contents in tissues was larger than that in development stages (**Figure 1B**). The differential statistical analysis identified that a large number of differential metabolites between the different tissues were also enriched in the metabolic pathway associated with AAAs and flavonoids (**Figure S1**). Specifically, the relative content of total flavonoids was higher in peel, while the relative content of AAAs was lower than that in both flesh and seed, especially at the mature stage (**Figures 1C–E**). In contrast, the relative contents of AAAs in flesh had the highest level, while that of total flavonoids had the lowest level at the mature stage (stage 4) (**Figure 1C**). From the general change in trend, the relative content of AAAs increased at mature stage (stage 4) in flesh and seeds (**Figures 1C**, **E**), while the total flavonoids decreased with all development stages in flesh and seeds. In peel, the relative content of total flavonoids was higher than that of AAAs at all development stages, respectively (**Figure 1D**). In addition, we also measured the absolute content of total flavonoids in *A. trifoliata* fruit. We did not detect flavonoids in mature flesh within the error range. In contrast, the flavonoid contents in the peel and seeds were measured to be 29.11 mg/g and 6.78 mg/g, respectively. In summary, these results suggested that an obvious inverse relationship existed between AAAs and their derivative flavonoids during the developmental process in *A. trifoliata* fruit.

### Identification of genes involved in AAA metabolic pathways at the genome level

A total of 104 genes putatively involved in AAA biosynthesis and metabolism pathways were identified in the *A. trifoliata* reference genome according to the KEGG database and previous (**Figure S2**). Among them, 17, 26 and 20 genes were further classified into AAA biosynthesis pathways, including the ‘shikimate’, ‘tryptophan biosynthesis’ and ‘arogenate and phenylpyruvate’ subpathways. Similarly, 14 and 27 genes were classified into AAA downstream metabolism pathways, including the ‘phenylpropanoid’ and ‘flavonoid’ biosynthesis subpathways, respectively (**Table S3**). Further synteny analysis found that 43 (41.3%) out of the 104 genes could be derived from WGD, and this proportion of WGD genes was higher than that 8329 of 24138 (34.5%) in the whole genome background (**Table S3**). This indicated that AAA metabolic pathways could be reinforced in the evolutionary process.

### Correlation analysis between gene expression and related traits of AAAs

**Figure 2:**
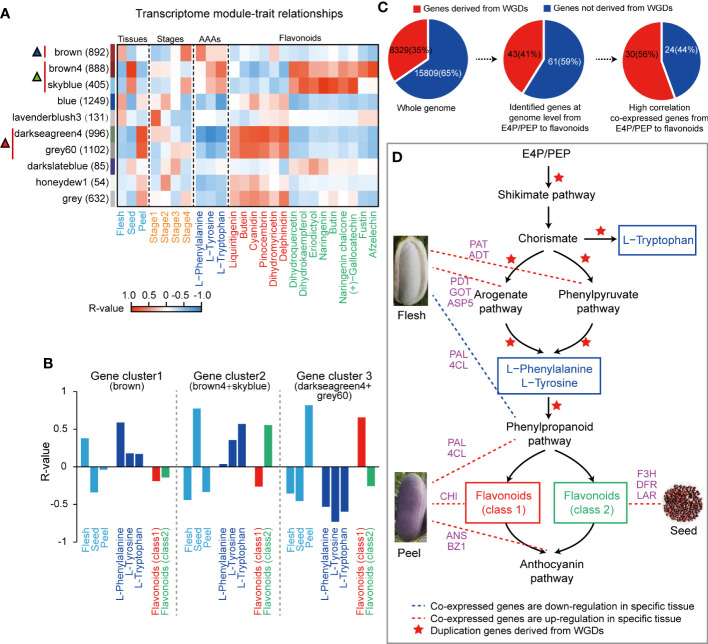
Aromatic amino acid biosynthesis and metabolism in *A. trifoliata*. **(A)** Heatmap correlation between the WGCNA gene set module and objective traits. The red vertical line and triangle marker represent the high positive module, and the numbers in brackets are the numbers of genes in each module. Flavonoids were divided into two classes by R-values. **(B)** Association of selected WGCNA modules with different tissues and metabolites. Mean R-values presented in the two integrated modules. **(C)** Percentage of genes derived from WGD events at different levels. **(D)** The postulated pathway for the biosynthesis of aromatic amino acids and flavonoids.

Transcriptome analysis of fruit samples was conducted to study the biosynthesis mechanism of AAAs. The correlation between the expressed gene cluster modules and traits including development stages, fruit tissues, AAAs and representative flavonoids was calculated by WGCNA method. A total of five highly correlated gene cluster modules were identified, which exhibited an inverse relationship between AAA and flavonoid content (**Figure 2A**). We further found that the coexpression correlation values between the ‘brown4’ and ‘skyblue’ modules (r= 0.76) and between the ‘darkseagreen4’ and ‘grey60’ modules (r= 0.95) were very similar, so we put the ‘brown4’ and ‘skyblue’ modules together as an integrated gene cluster 2, the ‘darkseagreen4’ and ‘grey60’ modules together as the other integrated gene cluster 3 in the following study (**Figure 2B**). In addition, the results of WGCNA showed that 15 flavonoids were clustered into two different classes: class 1 (liquiritigenin, butein, cyanidin, pinocembrin, dihydromyricetin and delphinidin) and class 2 (dihydroquercetin, dihydrokaempferol, eriodictyol, naringenin, butin, naringenin chalcone, (+)-gallocatechin, fustin and afzelechin) (**Figure 2A**). The expression of genes in gene cluster 1 of ‘brown’ module was positively correlated with AAAs, while negatively correlated with all flavonoids except dihydromyricetin in flesh, especially at stage 4; that in gene cluster 2 was highly positively correlated with Trp, Tyr and class 2 flavonoids, while highly negatively correlated with class 1 flavonoids in seed; and that in gene cluster 3 was highly negatively correlated with AAAs and class 2 flavonoids, while highly positively correlated with class 1 flavonoids in peel (**Figures 2A, B**). Obviously, the AAA metabolic pathway of *A. trifoliata* exhibited obvious tissue or fruit developmental stage specificity (**Figure 2B**). Then, we focused on the identification of the key genes involved in the metabolic pathway of both AAAs and flavonoids for the five modules.

### The evolutionary events mainly experienced candidate key genes involved in the AAA metabolism pathway

Among 104 annotated genes putatively involved in the AAA metabolism pathway, a total of 54 genes, consisting of 29 genes before AAA synthesis and 25 genes after AAA synthesis, were clustered into five highly correlated gene cluster module-trait modules, in which 8, 9, 2, 11 and 24 genes were clustered into three correlative gene clusters, respectively. Based on the results of AAA metabolism at the genome level and transcriptome and metabolome data in this study, we proposed a putative pathway from the tricarboxylic acid cycle to AAAs and then to flavonoids in *A. trifoliata* (**Figure S2**). Evolutionarily, 30 (56%) of 54 coexpressed genes experienced WGDs (**Figure 2C** and **Table S3**), in which 19 and 11 genes were putatively located before and after AAAs in the proposed pathways, respectively (**Figures S2**, **S3**). The proportion (66%) of coexpressed genes derived from WGD before AAAs was higher than that (44.0%) after AAAs in the proposed pathways (**Figure S2**). The results indicated that both the AAA and flavonoid biosynthesis pathways in *A. trifoliata* might simultaneously be reinforced by WGD events, but the degree of AAA biosynthetic processes could be stronger than that of flavonoids.

### Identification of key regulatory genes in AAA-related pathways

The reinforced AAA metabolic pathway exhibited obvious tissue or fruit developmental stage specificity (**Figure 2**). From the 54 coexpression genes on the putative pathway (**Figure 2D** and **Table S3**), there were five genes including *PAT* (*EVM0006124*), *ADT* (*EVM0001040*), *GOT2* (*EVM0008507* and *EVM0000841*) and *ASP5* (*EVM0012020*) in gene cluster 1 enriched in the arogenate and phenylpyruvate biological synthetic subpathways of both Phe and Tyr, six genes including three *PAL* (*EVM0013218*, *EVM0015158* and *EVM0002982*) and three *4-CL* (*EVM0023774*, *EVM0011793* and *EVM0005009*) encoding the limited enzymes of phenylpropanoid pathway and five genes *CHI* (*EVM0015959*), *ANS* (*EVM0023082*) and *BZ1* (*EVM0005744*, *EVM0012552* and *EVM0021545*) directly regulating class 1 flavonoids biosynthesis in gene cluster 3, and three genes including *F3H* (*EVM000232*3), *DFR* (*EVM0022942*) and *LAR* (*EVM0020322*) in gene cluster 2 directly regulating class 2 flavonoids biosynthesis, so that they were identified as the candidate genes that plays a key regulatory role in AAAs metabolic pathways (**Figure 2D** and **Figure S4**). In addition, among these five key genes, the *ADT* (*EVM0001040*) could encode an enzyme with a prephenate dehydratase (PDT) activity conferring (PAC) domain, the hallmark of PDT enzyme activity (**Figure S5**). This *ADT* (*EVM0001040*) was derived from WGD events.

### Identification and subcellular localization of hub genes

**Figure 3:**
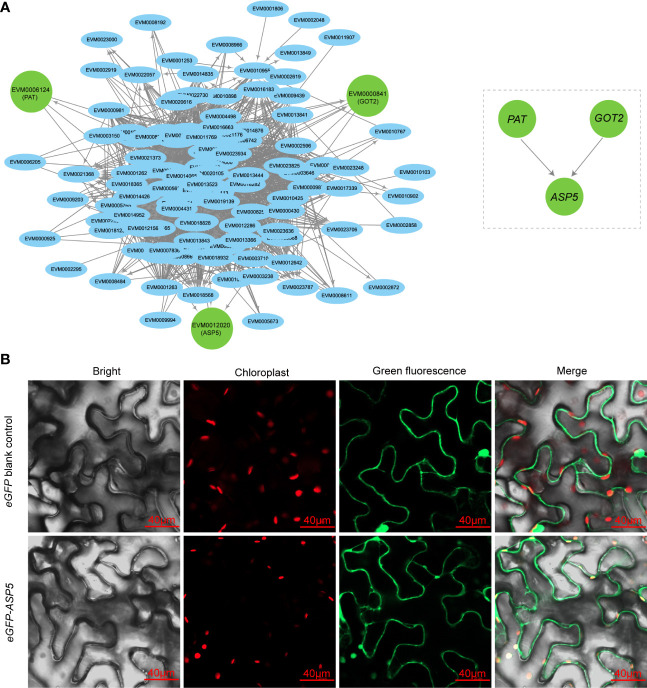
Subcellular localization of hub genes. **(A)** Hub genes of the aromatic amino acid biosynthesis pathway in the brown module, as represented by node and edge graphs. **(B)** Subcellular localization of *ASP5*. The *eGFP*-*ASP5* fusion plasmid was transiently expressed in *Nicotiana benthamiana* leaves. Plasmid only contain *eGFP* was also transiently expressed as control.

Hub genes have a high degree and importance of connectivity in biological interaction networks. We first screened three hub genes (*PAT*, *GOT2* and *ASP5*) by gene subnetwork analysis, and all three genes belonging to AAA biosynthesis were closely related to coexpressed genes in the gene cluster 3 and connected to several other genes with high connectivity within the network (**Figure 3A**). Interestingly, all three genes could encode aspartate aminotransferase, a very important enzyme for biosynthesis of Phe and Tyr but have differences in the subcellular location of expression. The *PAT* was probably cytoplasmic, *GOT2* was probably mitochondrial, and *ASP5* was probably located in the chloroplast (**Table S3**). In the network, both *PAT* and *GOT2* were directly connected with *ASP5* genes, while the direct connection between *PAT* and *GOT2* was obviously absent (**Figure 3A**), so *ASP5* was treated as the central hub gene in the key node and could play a pivotal role in the regulatory network. Finally, the transient expression of *ASP5* in *N. benthamiana* leaves further confirmed that *ASP5* was localized in the chloroplast (**Figure 3B**), in which the key node of biosynthesis of both Phe and Tyr was present.

## Discussion

Humans survived and profited from the exploitation of plant resources as their final and main food source. In the past, especially in remote antiquity, people only considered macronutrients, mainly carbohydrates, proteins and fats, when they found new food sources and largely ignored micronutrients, such minerals and vitamins, which resulted in a number of diseases threatening our health and lives. Today, both the components and contents of natural beneficial materials in food have received increasing attention. Therefore, finding new types of food from various plants, especially multipurpose functional plants, is valuable and significant work.

The fruit of *A. trifoliata*, also called ‘augmelon’, ‘bayuegua’ and ‘wild bananas’, is a new fresh edible fruit, in which the flesh, as the part to directly eat, is rich in vitamins, minerals, free amino acids and proteins (Guan et al., 2022). We found that the total free amino acid content of *A. trifoliata* was the highest among the five tested fruit varieties and further found that the contents of both total AAAs (Trp, Phe and Tyr) were significantly higher than those of the other four popular fruits (**Table 1**). In addition, in all five fruit varieties, Trp was the most abundant AAA, and Phe was the least abundant AAA, except in grapes. The results indicated that the metabolic pathways of *A. trifoliata* could be different from those of the other four common fruits, and this difference could be explained by evolutionary processes. AAAs are important components of proteins in all organisms. In fact, AAAs are also essential materials for the survival of many animals, including humans, although Tyr can be synthesized from Phe by Phe hydroxylase in animals (Maeda and Dudareva, 2012) because animals derived from fishes have lost the ability to synthesize aromatic amino acids using common substances (Costa et al., 2015). In addition, AAAs, especially Phe, also serve as precursors of various secondary metabolites of plants (Dodd et al., 2017). Hence, the abundant AAA contents in the fruit of *A. trifoliata* provide a great opportunity to further understand the metabolic mechanism of AAAs and to agriculturally exploit new fresh fruit crops from wild plant resources.

We identified a total of 581 metabolites in different fruit tissues and developmental stages (**Figure 2A**, **Table S2**), in which flavonoids not only had the largest number of categories (**Figure 2A**) but also exhibited tissue specificity (**Figure 2**). The content of flavonoids was not detectable in ripe flesh, while it was up to 29.11 mg/g in ripe peel, which usually resulted from gene tissue-specific expression rather than the difference at the genome level. The tissue-specific characteristics of metabolites such as flavonoids provide reasonable explanations for the long history as medicinal plants and good environmental adaptation of *A. trifoliata* (Li et al., 2010). Moreover, the obvious inverse relationship between AAA and flavonoid contents also indicated that both AAA synthetic pathways and catabolic pathways could be simultaneously reinforced in evolutionary processes. In addition, we noticed that the derivatives of AAAs were mainly produced by the pathway with the precursor of Phe and Tyr rather than Trp (**Table S2**), which suggested that the biosynthesis of Trp was reinforced rather than downstream catabolism.

WGD was an important evolutionary force in the plant, and several biological processes, enzymes and transporters in some specific metabolic processes were preferentially reserved after whole-genome triplication of eudicots called ‘γ’ or independent WGDs around the Cretaceous/Paleogene (K-Pg) boundary (Wu et al., 2020). The shikimate pathway derives AAAs and some eudicot-specific secondary metabolites, such as gallo- and ellagitannins (Okuda et al., 2000; Muir et al., 2011), in which more than 30% of photosynthetically fixed carbon is directed (Tohge et al., 2013), while the phenylpropanoid pathway is one of the most extensively studied specialized metabolic routes, which sits on the boundary between primary and secondary metabolism (Dong and Lin, 2021). In this study, flavonoids were the most abundant, both in number of flavonoids and contents (**Table S2**). Therefore, we paid much attention to the number and proportion of genes experiencing WGD events in both the shikimate and phenylpropanoid pathways and in the synthetic processes of both AAAs and flavonoids (**Figures 2D** and **S2**). We found that the proportion of genes derived from WGD and involved in AAA metabolism pathways was 41.3% (43/104), which was higher than the 34.5% (8329/24138) observed in the whole genome (**Table S3**). Among 54 coexpressed genes, 30 (56%) experienced WGDs (**Figures 2C** and **S3**), and further comparison found that the proportion of genes derived from WGD in the synthetic and catabolic (the main synthesis of flavonoids) pathways of AAAs was 66% (19/29) and 44.0% (11/25), respectively. Moreover, many of these genes exhibited coexpression with 15 major metabolic node flavonoids and both Phe and Tyr. The derivatives of Trp had fewer categories and lower contents, while Trp had the highest content compared with Phe and Tyr, so we would suggest that both the synthetic pathway of all three AAAs and only the catabolic pathway of Phe were reinforced by WGD, which could be an evolutionary genomic basis for the rich free AAA content in the flesh of *A. trifoliata*.

Many studies confirmed that the biosynthetic flux of both Phe and Tyr is mainly through the arogenate pathway located in the chloroplast and is largely regulated by the arogenate dehydratase or prephenate dehydratase (Qian et al., 2019), however, several species, such as *Arabidopsis thaliana* (Bross et al., 2011; Westfall et al., 2015), *Petunia hybrida* (Yoo et al., 2013) and *Pinus pinaster* (El-Azaz et al., 2016), can sufficiently synthesize them through the phenylpyruvate pathway and are mainly regulated by the microbial-like PDT enzyme (El-Azaz et al., 2016). In this study, five genes (two *GOT2*, one *PAT*, one *ADT* and one *ASP5*) in the gene cluster 3 simultaneously took part in both the arogenate and phenylpyruvate pathways of AAA biosynthesis (**Figure 2**), which indicated that *A. trifoliata* could synthesize Phe and Tyr by both the arogenate and phenylpyruvate pathways. One *ADT* (*EVM0001040*) of the five key regulatory genes in the gene cluster 3 contained a PAC activity domain (**Figure S5**), which was the hallmark domain of the PDT enzyme, so we would like to accept that *A. trifoliata* could have the other complementary synthetic pathway of Phe and Tyr through the phenylpyruvate pathway (**Figure 2D**). Interestingly, *EVM0001040* was derived from WGD, affording a chance for the duplicated gene to functionally differentiate. In addition, we further found that all three hub genes (*PAT*, *GOT2* and *ASP5*) in the network putatively encoded aspartate aminotransferase (**Figure 3A**). Among the three hub genes, *ASP5* could play a more important role in regulating the synthesis of both Phe and Tyr because there was a direct connection between *ASP5* and both *PAT* and *GOT2*, while there was no direct connection between *PAT* and *GOT2*. Moreover, *ASP5* was also localized in chloroplasts (**Figure 3B**), in which the arogenate pathway is located. The results suggested that the main arogenate pathway of both Phe and Tyr synthesis in *A. trifoliata* had a stronger regulating ability compared with other fruit varieties. Therefore, the possibility that *A. trifoliata* could have the phenylpyruvate pathway of both Phe and Tyr biosynthesis was further increased in our opinion.

Likewise, the catabolism of AAAs, especially Phe, was our last concern because it would be the other important factor affecting the accumulation of AAAs and their derivatives in various tissues of *A. trifoliata* fruit, which was largely regulated by limited enzymes encoded by *PA*L and *4-CL* genes (Barros and Dixon, 2020). In our study, three *PAL* and three *4-CL* coexpressed genes were identified in the gene cluster 2 by WGCNA, and many (66.7%) of them were also duplicated by WGD events (**Table S3**). The downstream diversifying branch pathways of the phenylpropanoid pathway could be a good explanation for the difference in AAA and derivative contents in various fruit tissues (**Figure 2** and **Figure S4**), which could be regulated by gene tissue-specific expression. For example, the high AAA content in flesh possibly resulted from the blockage of the phenylpropanoid pathway, while the low AAA content in peel and seed could be associated with the enhanced synthetic pathway of class 1 and class 2 flavonoids, respectively.

## Conclusion

Comprehensively, we found that the AAA content in the flesh of *A. trifoliata* fruit was significantly higher than that in the flesh of the other four fruits (apple, pear, banana and group), and there was an inverse relationship between the contents of AAAs and their derivative flavonoids in various tissues of *A. trifoliata* fruit. Further multiomics analysis revealed that the evolutionarily reinforced synthetic pathway of all three AAAs, the largely regulating ability conferred by *ASP5* (*EVM0012020*) in the arogenate pathway and the complementary phenylpyruvate pathway endorsed by *ADT* (*EVM0001040*) of both Phe and Tyr biosynthesis afforded reasonable explanations for the high AAA content in the flesh of *A. trifoliata* fruit. Likewise, gene-specific expression could be the main reason for the inverse relationship between AAAs and flavonoids. The results will be helpful to completely understand the metabolic mechanism of AAAs in plants and valuable to commercially accelerate the exploitation of *A. trifoliata* as a fresh fruit crop and medicinal plant by molecular breeding strategies.

## Data availability statement

The raw sequencing data presented in the study are deposited in the National Centre for Biotechnology Information (NCBI) with accession numbers from SAMN16551931 to SAMN16551942 under the BioProjectID PRJNA671772. Other original contributions are included in the article/**Supplementary Material**. Further inquiries can be directed to the corresponding author.

## Author contributions

SZ: formal analysis, Software, Writing - Original Draft, Visualization, Methodology. JG: Validation, Investigation, Resources, Writing - Original Draft. CC: Data Curation, Formal Analysis. FT: Investigation, Resources. PL: Conceptualization, Writing - Review and Editing, Project Administration, Funding Acquisition. All authors contributed to the article and approved the submitted version.

## Funding

This work was supported by grants from the Science and Technology Department of Sichuan Province, China (2020YFN0091 and 2020YJ0331).

## Conflict of interest

The authors declare that the research was conducted in the absence of any commercial or financial relationships that could be construed as a potential conflict of interest.

## Publisher’s note

All claims expressed in this article are solely those of the authors and do not necessarily represent those of their affiliated organizations, or those of the publisher, the editors and the reviewers. Any product that may be evaluated in this article, or claim that may be made by its manufacturer, is not guaranteed or endorsed by the publisher.
