# Supplementary material for: Multiomics analysis elucidated molecular mechanism of aromatic amino acid biosynthesis in Akebia trifoliata fruit
Source: Front Plant Sci. 2022 Nov 8;13:1039550. doi: 10.3389/fpls.2022.1039550 (PMC9680012; doi:10.3389/fpls.2022.1039550)
Supplement: Supplementary file 2 [file DataSheet_2.docx]

**Supplementary materials**


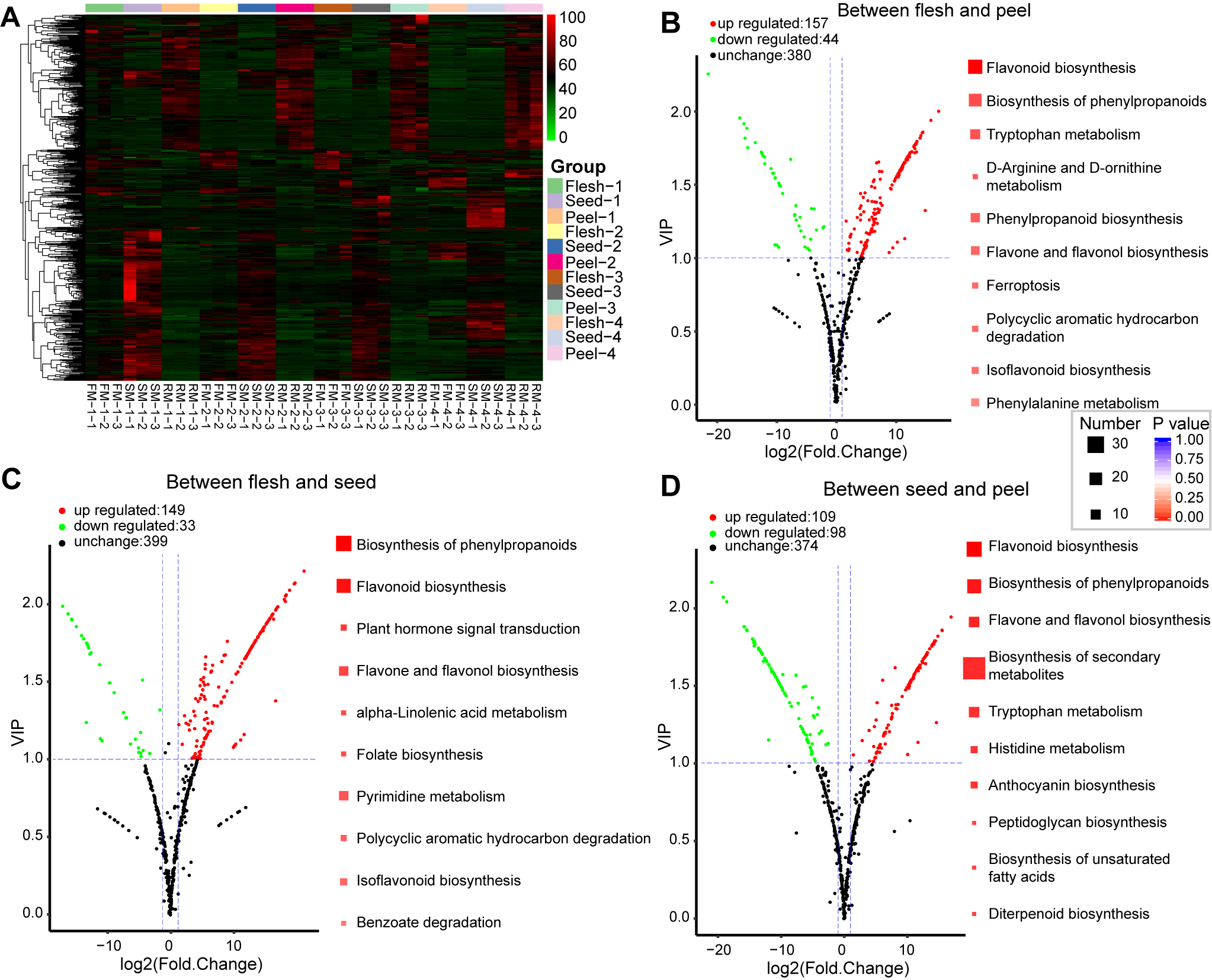


**Fig. S1.** Analysis of fruit metabolomics. (A) Cluster results of 581 metabolites. (B-D) KEGG enrichment of differential abundant metabolites among the 3 tissues. The differential metabolites were identified with a threshold of fold change (FC) ≥ 2 or FC ≤ 0.5 and a variable importance in project (VIP) value ≥1. The square next to the volcano plot represent the number of metabolites enriched in each pathway, only top 10 differential metabolites which ordered by P value from top to bottom were shown.


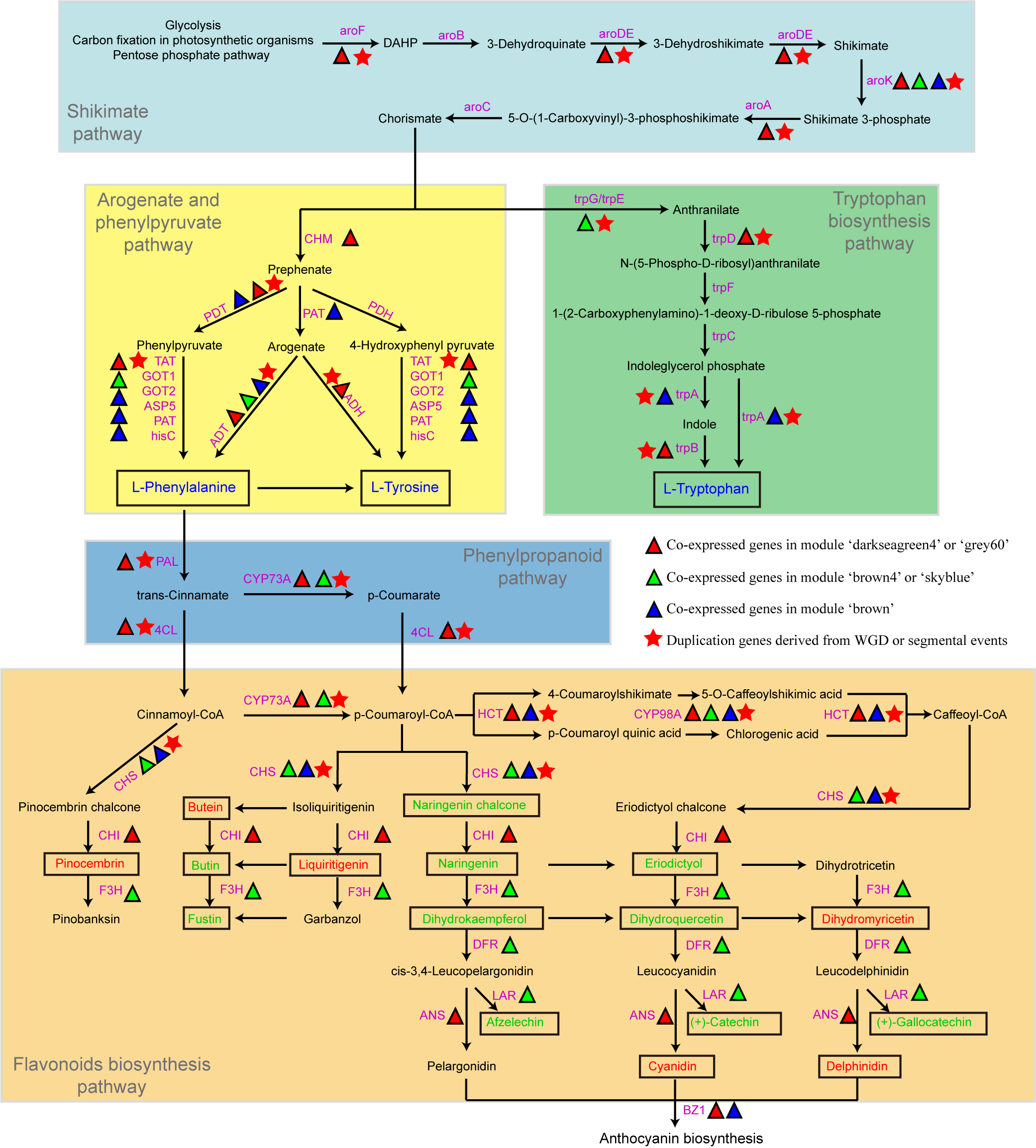


**Fig. S2.** Model of an integrated AAA and flavonoid biosynthesis pathways with coexpressed genes. Black arrows are known interactions, gene names are shown in purple, AAAs are shown in blue, and two classes of flavonoids are shown in green and orange.


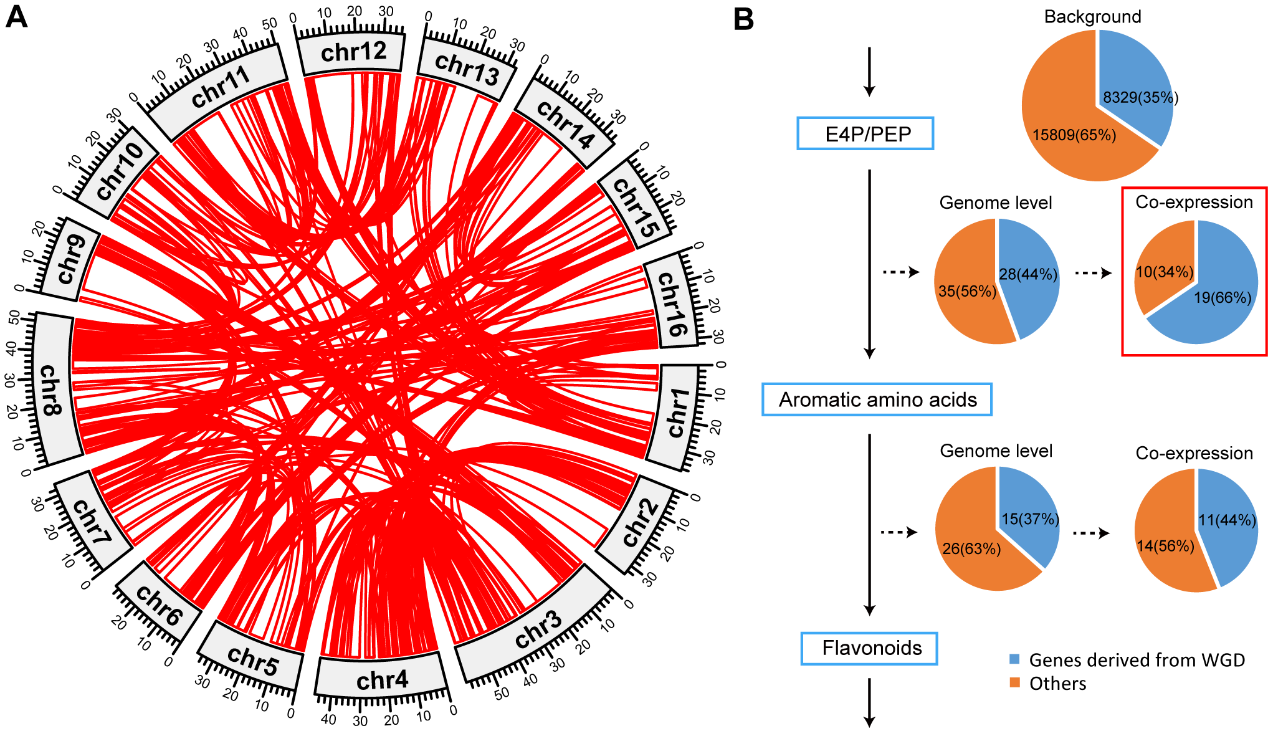


**Fig. S3.** Statistics of genes derived from whole genome duplication events involved in AAA biosynthesis. (A) Synteny analysis of the *A. trifoliata* genome. Red lines represent the collinearity blocks, and 8329 out of 24138 genes were in these synteny blocks. (B) Percentage of genes in AAA-related pathways derived from WGD events at different levels. Highly correlated c-expression genes were identified by WGCNA.

**
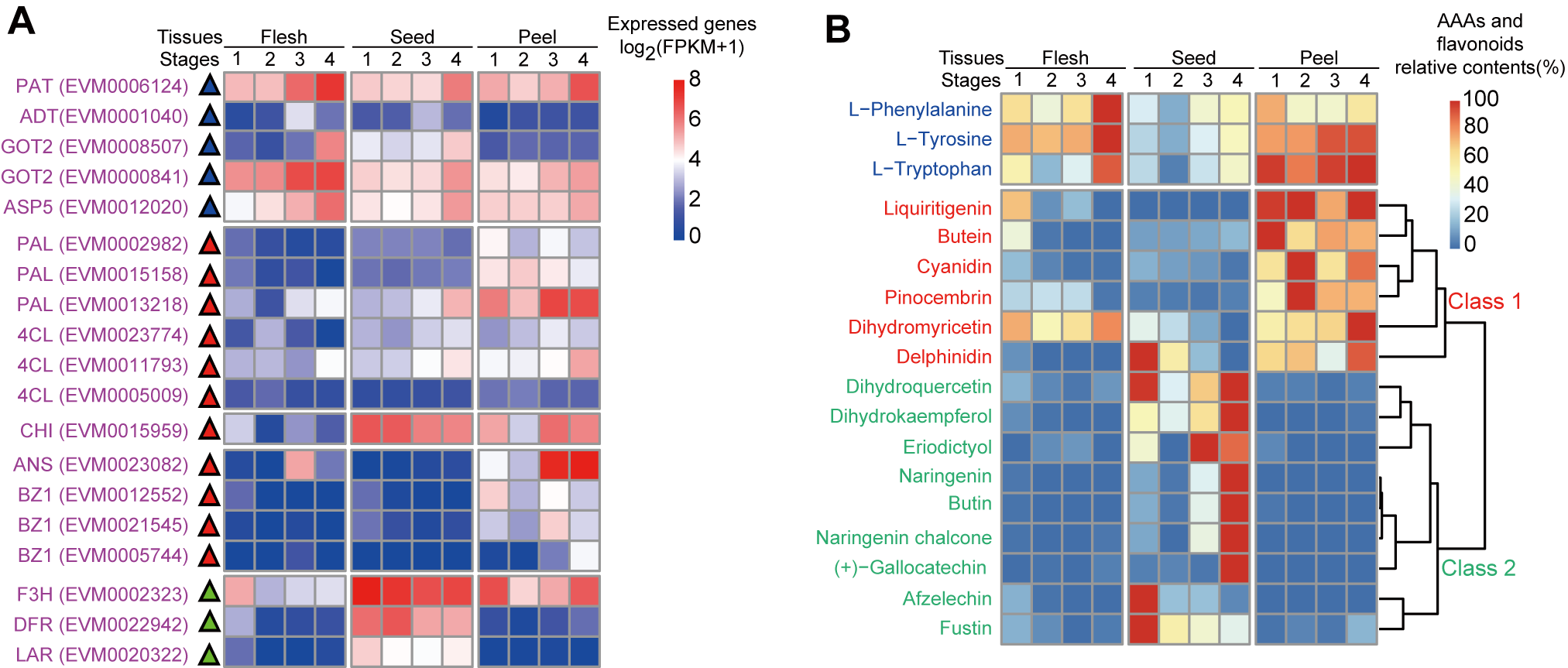
**

**Fig. S4.** Heatmap of key gene expression levels and metabolite contents. The flavonoids were clustered into two class groups by TBtools.


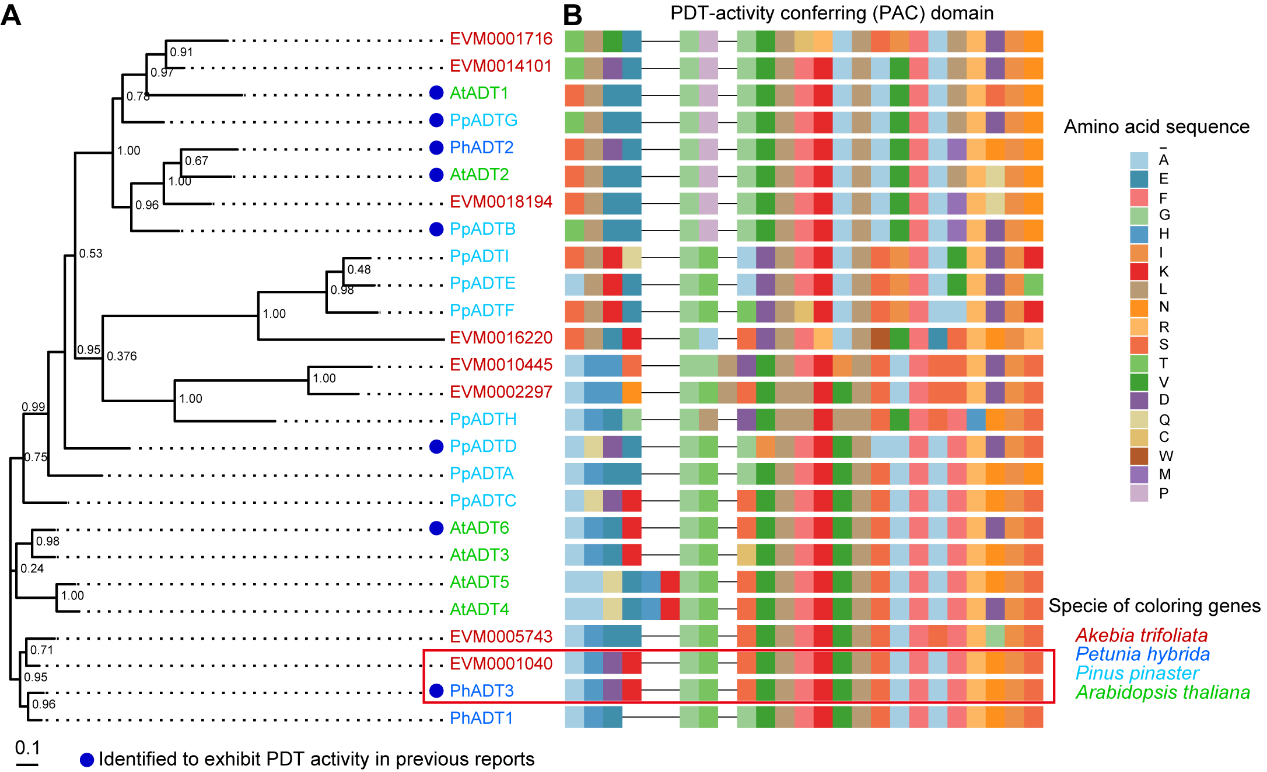


**Fig. S5.** Functional identification of enzymes with prephenate dehydratase (PDT) activity in *A. trifoliata.* (A) Maximum likelihood tree of arogenate dehydratase (*ADT*) genes in *A. trifoliata*, *P. hybrida*, *P. pinaster* and *A. thaliana*. (B) Multiple sequence alignment of the PDT activity conferring domain.
